# Tracking Subjective Sleep Quality and Mood With Mobile Sensing: Multiverse Study

**DOI:** 10.2196/25643

**Published:** 2022-03-18

**Authors:** Koen Niemeijer, Merijn Mestdagh, Peter Kuppens

**Affiliations:** 1 Faculty of Psychology and Educational Sciences Katholieke Universiteit Leuven Leuven Belgium

**Keywords:** mobile sensing, sleep, subjective sleep quality, negative affect, depression, multiverse, multilevel modeling, machine learning, mood, mood disorder, mobile sensors, sleep quality, clinical applications

## Abstract

**Background:**

Sleep influences moods and mood disorders. Existing methods for tracking the quality of people’s sleep are laborious and obtrusive. If a method were available that would allow effortless and unobtrusive tracking of sleep quality, it would mark a significant step toward obtaining sleep data for research and clinical applications.

**Objective:**

Our goal was to evaluate the potential of mobile sensing data to obtain information about a person’s sleep quality. For this purpose, we investigated to what extent various automatically gathered mobile sensing features are capable of predicting (1) subjective sleep quality (SSQ), (2) negative affect (NA), and (3) depression; these variables are associated with objective sleep quality. Through a multiverse analysis, we examined how the predictive quality varied as a function of the selected sensor, the extracted feature, various preprocessing options, and the statistical prediction model.

**Methods:**

We used data from a 2-week trial where we collected mobile sensing and experience sampling data from an initial sample of 60 participants. After data cleaning and removing participants with poor compliance, we retained 50 participants. Mobile sensing data involved the accelerometer, charging status, light sensor, physical activity, screen activity, and Wi-Fi status. Instructions were given to participants to keep their smartphone charged and connected to Wi-Fi at night. We constructed 1 model for every combination of multiverse parameters to evaluate their effects on each of the outcome variables. We evaluated the statistical models by applying them to training, validation, and test sets to prevent overfitting.

**Results:**

Most models (on either of the outcome variables) were not informative on the validation set (ie, predicted *R*^2^≤0). However, our best models achieved *R*^2^ values of 0.658, 0.779, and 0.074 for SSQ, NA, and depression, respectively on the training set and *R*^2^ values of 0.348, 0.103, and 0.025, respectively on the test set.

**Conclusions:**

The approach demonstrated in this paper has shown that different choices (eg, preprocessing choices, various statistical models, different features) lead to vastly different results that are bad and relatively good as well. Nevertheless, there were some promising results, particularly for SSQ, which warrant further research on this topic.

## Introduction

### Background

Sleep is important for well-being and plays a key role in people’s mood and their risk for mental health problems such as depression. Therefore, obtaining a reliable picture of people’s sleep quality critical for the study, prevention, and treatment of mood disorders. However, most of the existing methods are based on obtrusive techniques, either asking participants to wear dedicated technological devices (in the laboratory or otherwise) or to self-report their sleep patterns in a sleep diary. Therefore, both methods are burdensome for individuals and are not appropriate for long-term monitoring. If a method that is capable of unobtrusively tracking a person’s sleep quality is developed, it would mean an important leap forward. One potential solution is the use of smartphones that have become ubiquitous in today’s society. Approximately 76% of adults in countries with advanced economies have a smartphone [1], a device equipped with several sensors that automatically record user behaviors, some of which may be considered sleep-related ones. This study aims to examine to what extent automatically recorded information with smartphones—known as mobile sensing—can be used to predict self-reported subjective sleep quality (SSQ), mood, and the risk of mood disorders in the form of depression.

Sleep quality can be measured either in terms of subjective, self-reported sleep quality or can be captured with objective measurements such as polysomnography (PSG) and more recently actigraphy. Subjective and objective sleep quality are only weakly related [2-4] because they measure different aspects of sleep quality, namely physical sleep quality (objective) or a person’s perception of sleep quality (subjective). However, they are both predictive of moods [5-7] and mood disorders [8-10]. Given the obtrusiveness of objective measurement methods and their adversity to use over long periods, we focus on predicting SSQ. Therefore, the primary outcome of this study is to measure SSQ with mobile sensing. As a secondary outcome, given the relevance of sleep quality for moods and mood disorders, we will examine to what extent mobile sensing is capable of predicting moods in terms of the daily negative affect (NA) and risk for mood disorders in terms of depression symptom severity. Thus, the underlying mechanism (and the underlying data on which this mechanism relies) is the relation between sleep and mental health.

### Related Work

In most studies, an accelerometer is used to detect smartphone movements that are seen as synonymous with participant movements in bed [11-14]. Such movement detection is also used in commercial sleeping apps, such as Sleep as Android [15], Sleep Cycle [16], and Apple’s native health app. Accelerometers can be used for movement detection with and without instructions to the participants. For example, researchers can instruct participants to place their smartphone next to or under their pillows. However, for the sake of ecological validity, this is usually not recommended. Another seemingly straightforward feature indicating sleep is whether the smartphone is being used (ie, screen on/off events) [14,17]. These screen events have proved useful for detecting circadian rhythms and people’s temporal sleep preferences [18]. A more contextual feature comes from the light sensor; ambient light indicates whether a room is dark and thereby helps determine whether a participant is sleeping [12,19,20]. Similarly, a microphone can pick up ambient sound, thereby confirming whether a participant is in a quiet environment [11,14,20].

Early efforts were made, for example, using location (GPS), contextual (sound and light), physical activity (accelerometer), and communication data (text and call logs) to infer SSQ measured with the Pittsburgh Sleep Quality Index [21], divided into 4 categories [22]. Using these features in a factor graph model, an accuracy of 78% was achieved. Other pioneering research on mobile sensing and sleep was conducted by comparing the quality of a smartphone accelerometer with an actigraph accelerometer for sleep monitoring [23]. Both devices showed reasonable agreement with all features except sleep onset latency. However, on a more critical note, 1 study [24] found no significant correlation between sleep measured by PSG and a smartphone app, although the mobile app did have high sleep-wake detection performance (85.9%) in relation to PSG. Thus, although mobile sensing for sleep detection has attained modest success, it is unclear how robust these methods are to changes in features and how well they describe sleep in comparison to PSG.

Despite PSG being considered the gold standard for sleep research, it is being recently rivaled by numerous wearable devices that feature an integrated accelerometer (among other sensors). Moreover, a mobile app was developed that achieved better user experience and lower perceived intrusiveness than wearables [11]. Using light, phone usage (including screen state), physical activity, and sound features in a completely “hands-off” approach, the app had an estimated sleep duration error of approximately 42 minutes, a slightly worse result than the other sleep detection methods, with 23 minutes when the smartphone is on the bed and 10 minutes for wearables. Subsequently, an app called StudentLife was developed with which a significant negative correlation among sleep, depression, and stress was found [19]. By using the same features as those in an earlier study [11], 95% of bedtimes were predicted with an error of 25 minutes from the ground truth as measured with an actigraph (Jawbone UP). Bringing the sensing accuracy of smartphones on par with that of wearables is significant because wearables are still not pervasive, thus making smartphones the least obtrusive solution for now.

A more indirect way of assessing sleep is by dividing a night into small epochs and predicting whether a participant is asleep or awake through self-reported bed and wake-up times or actigraphy. Of course, these sleep-wake states can then be used to derive other features that may subsequently be used for predicting sleep proxy measures. For example, 10-minute segments of sleep-wake states were predicted with 93% accuracy, but daily sleep quality was predicted with 84% accuracy [12]. Similarly, 15-minute bins were predicted with 89% accuracy using only screen on/off events [25]. Furthermore, 88.8% accuracy was reported when determining whether a 10-minute segment was a sleep state, whereas using the time of day alone achieved 86.9% accuracy [26]. Moreover, the accuracy per participant ranged from 65.1% to 97.3% although lower accuracies could mostly be attributed to missing data reports and misreports. After adjustments, the accuracy was increased to 91.8%, corresponding to a median absolute deviation (MAD) of 38 minutes for the sleep start time and 36 minutes for the sleep end time.

### This Study

We analyzed data from a 2-week study involving various smartphone sensors and the experience sampling method (ESM, also known as ecological momentary assessment) to collect SSQ in the morning and daily moods in terms of NA, as well as a 1-time assessment of person-level depression using a validated depression screening instrument. As mentioned above, several theoretically and empirically based predictions can be made regarding what sensors are the most significant for predicting SSQ and the associated variables of daily moods and depressive symptom severity. In addition, there are multiple ways to process the data based on different thresholds and data combinations, and there are different options to model the relationship between mobile sensing and the predicted variables. To examine this variability, we perform a multiverse analysis [27] in which we compare prediction results across multiple sensors, extracted features, thresholds, outlier methods, and prediction models. In other words, essentially all sensible combinations of these aspects, choices referred to as multiverse parameters*,* are evaluated in terms of how they are capable of predicting the outcome measures. Using this approach, this study not only aims to predict sleep with mobile sensing but also to investigate (1) which model, (2) which feature, and (3) which sensor works best within the context of the study. Consequently, this method is an analysis of robustness and transparency because it shows how different choices affect the outcome.

## Methods

### Overview of Data Preprocessing

As already mentioned earlier, we focused on the many seemingly arbitrary choices to be made during data preprocessing. Consequently, we performed a multiverse study showing the robustness of these choices. Following this approach, this section describes each aspect of the multiverse approach separately. First, various preprocessing steps were conducted for minimizing the number of participants with faulty data, namely removing participants with fewer than 10 data points for a sensor, sensors where all values were 0 (broken sensor), and 1 participant who had 40,000 times more data points than the participant with the second highest number of data points. In this regard, we present preprocessing of the different sensors in this study and describe when they are considered to indicate that a participant is sleeping, often having more than 1 threshold. Next, we discuss how features were built. These features are used in various statistical models. In the context of a multiverse approach, we experimented with several models to see which one worked best. Finally, we tried different outlier removal methods to catch faulty data entry and unlikely data instances.

### Data Collection

Data were collected during a 2-week study in 2018 [28]. Participants installed 2 separate apps on their smartphones, 1 for collecting sensor data and 1 for tracking their moods and SSQ using ESM. Mobile sensing data were gathered from specific (software) sensors to account for behavioral and contextual factors. These sensors are described in Table 1. Participants were instructed to charge their smartphones at night and connect to Wi-Fi whenever possible. On the other hand, data on the participants’ SSQ and NA were collected using ESM, where they were prompted 10 times per day with a 16-item questionnaire measuring, among other parameters, their momentary (positive or negative) affect and how they slept if it was their first survey of the day. Depression levels were recorded only after the study using the Depression, Anxiety and Stress Scale questionnaire [29,30]. Katholieke Universiteit  Leuven’s Social and Societal Ethics Committee, whose directives are based on the Helsinki Declaration, approved the study (reference no. G-2018 01 1095). Written electronic consent from all subjects included in this study was obtained.

In total, 230 people responded to the participant selection questionnaire posted on social networking sites and other places frequented by students at the University of Leuven. Participants were excluded if they (1) did not understand Dutch, (2) did not have sufficient activity on their devices, (3) did not own an Android smartphone (except Huawei, Wiko, Medion, or Xiaomi), and (4) did not grow up with smartphones (ie, below 32 years of age). Among the 114 individuals who participated in an information session, 69 decided to join; 2 refused to sign the informed consent, and app installation failed for another 2 individuals. After excluding 5 participants who completed fewer than 30 ESM surveys, 60 participants remained. Furthermore, all data were preprocessed such that participants with fewer than 10 observations in total or only 0s (indicating a broken sensor) were left out. Single observations were left out if they were far away in time from any other observations (ie, if there was a gap in time in the data) using boxplot outliers. Next, values for each sensor were visually inspected to exclude participants who had very poor data quality. Participants were only retained if they had sufficient data for all sensors (ie, more than 10 observations), resulting in a final data set of 50 participants. Because participants were mainly university students and advertising was mainly done on social media and the campus of the faculty of psychology, the majority of the sample was young and female (14 male and 36 female aged 17-32 years, mean 21*.*90, SD 2*.*38 years).

### Sensor Data Preprocessing

In this study, we used the following sensors: accelerometer, charging status, light sensor, Android activity recognition (AAR), screen activity, and Wi-Fi status. Table 1 gives an overview of the sensors and how their data were handled to obtain information on participants’ sleep quality.

**Table 1:** Description of sensors used in this study^a^.

Sensor	Description	Considerations	Indication of sleeping when...
Accelerometer	Acceleration in m/s^2^ along the x-axis	Other axes (y and z) not measured owing to programming error	Absolute value is less than (0.25, 0.5, 1) m/s^2^.
Charging	Indication of whether the smartphone is charging As participants were instructed to leave their smartphone charging at night, a charging smartphone can mean that the participant is sleeping.	Momentary sleep interruptions are unlikely to be picked up by this sensor.	Battery is charging.
Light	Illuminance in lux^b^ captured by the light sensor (usually at the front-top side of the smartphone)	Room with translucent curtains may simulate a wake-up state. Smartphone may be upside down.	Value is less than (8, 12, 16) lux.
Physical activity	Category of physical activity as measured by AAR^c^ (ActivityRecognitionApi app)	Proprietary algorithm obfuscates how AAR is transformed into discrete activities.	AAR returns “Still.”
Screen activity	Logs whether the screen has been changed to on or off.	Duplicate entries (screen off multiple times without screen on) are frequent. There is no distinction regarding whether the app turned the screen on (eg, notifications) or the participants.	Screen is off.
Wi-Fi	Information about whether the smartphone is connected to Wi-Fi	Unlikely to pick up temporary wake-up states	Connected to Wi-Fi.

^a^ The considerations column discusses issues that are (likely) associated with the sensors or their implementation. The sleeping when... column indicates when a sensor decides that a participant is sleeping in a window. Multiple values in parentheses indicate that the same data have been used but with a different cutoff value.

^b^lux is the SI unit for intensity.

^c^AAR: Android activity recognition.

One of the core elements in this multiverse study is inspecting each sensor and applying suitable preprocessing. The accelerometer was thought to be highly indicative of the sleep state [14,20]. However, as this sensor was sampled only every 2.9 minutes on average (SD 6*.*72 minutes with the longest interval being 15 minutes), only movement at that specific moment leads to the detection of a nonsleeping state. Additional problems with this sensor were that owing to a programming error, only the x-axis data were collected and the sensor in the participants’ smartphones was uncalibrated. The latter issue led to some accelerometers not showing 0 acceleration at their lowest point. To offset this, accelerometer values were centered per participant, and the absolute values were calculated subsequently, such that a corresponding sleep or wake state could be derived from the threshold values specified in Table 1. As the accelerometers were not calibrated beforehand, we attempted to align them by centering. Moreover, for every acceleration, there must be an opposite deceleration. Physical activity is the product of on-the-fly processing by Google’s AAR [31]. In brief, AAR runs an algorithm that combines multiple sensors to form discrete activities. In this research, only the activity “Still” was used because this indicates that the smartphone is not moving (whereas the other activities indicate some type of movement).

The screen state software sensor was preprocessed in such a way that interactions of less than 12 seconds were ignored. This is necessary because some of the smartphone screens light up whenever there is a new notification and thus pollute the actual stream of the screen state. On the other hand, if people use their smartphone in the middle of the night for, say, checking the time, this will most likely also be thrown out. For the charging, light, and Wi-Fi sensors, no further preprocessing was applied.

After cleaning the data, the participant’s nights were divided into windows of 5 minutes. To avoid a high number of false positives, only data from 10 PM to 10 AM on the next day were considered. For each window and sensor, a majority vote determined whether a participant was sleeping. For example, if the app sensed that the smartphone was charging twice and that it was not charging once (within the same window), a majority vote set the window to sleeping. If there were no data in the window, the last observations (ie, of the previous window) were carried forward.

### Feature Creation

**Figure 1:**
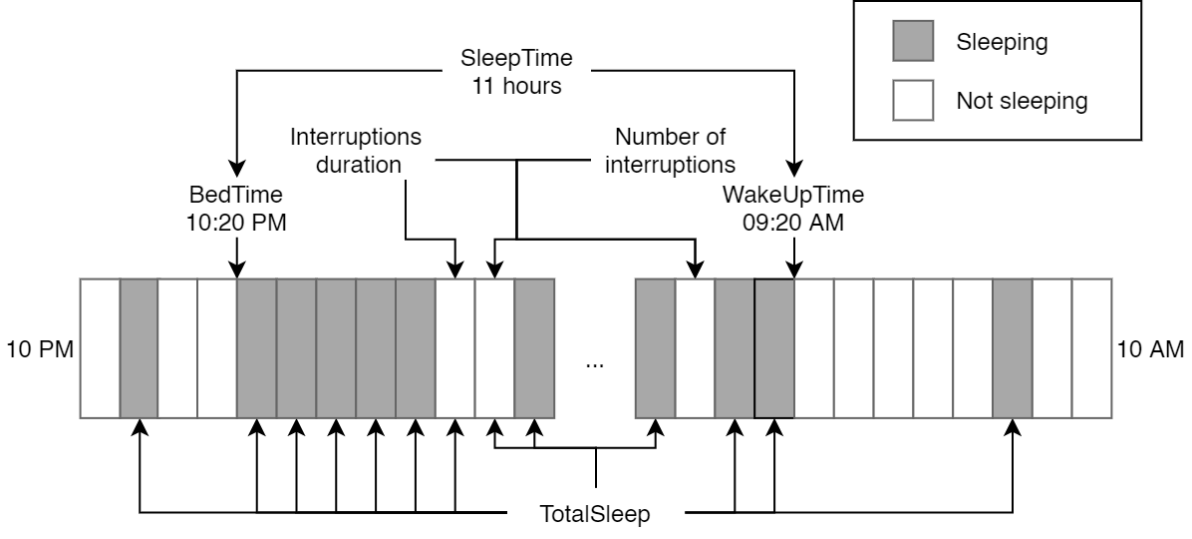
A fictional example of the features used in this study. This figure only depicts the first and last hours of the windows for illustration purposes, where the … obscures the rest of the night. Gray bars indicate sleep states, and white ones indicate nonsleeping states. The night starts at 10:20 PM (ie, before the first sleeping window) because BedTime requires 3 to 5 adjacent windows (4 windows in this example). Similarly, WakeUpTime starts at 9:20 AM because this denotes the start of 3 to 5 adjacent windows, depending on the chosen parameters. Notice how SleepTime is the time between BedTime and WakeUpTime, whereas TotalSleep is simply the sum of all the sleeping windows. Finally, the number of interruptions is given as the number of nonsleeping sequences minus the number of sleeping sequences; the interruptions duration feature includes all the nonsleeping windows within SleepTime.

Several features were extracted from the data that were deemed indicative of sleep quality. Figure 1 displays a visual example of all the features explained in this section. The basic building blocks for most features are the BedTime and WakeUpTime features that are found by examining 3 to 5 adjacent windows where a participant is sleeping (BedTime) or not sleeping (WakeUpTime) according to a sensor. Concretely, for the first 3 to 5 adjacent windows where a participant is sleeping, the first window of that sequence is the BedTime window. The opposite is true for WakeUpTime, namely the first window of the first 3 to 5 adjacent windows where a participant is not sleeping.

From the initial features of BedTime and WakeUpTime, several others could be calculated as well. First, SleepTime was calculated as the time between BedTime and WakeUpTime. Second, sleep quality may be impacted when a participant sleeps unusually longer or shorter than usual. To capture this, we computed the deviation from each participant’s average BedTime and WakeUpTime (in hours) for each night (abbreviated as devAvgBedTime and devAvgWakeUpTime, respectively). Hours have been recentred such that 10 PM=0*,*11=1*...,*10=12. Third, the number and duration of nightly interruptions (abbreviated as nInterruptions and InterruptionsDuration, respectively) are calculated as follows: Every pattern of neighboring wake-up minus the sleeping windows counts as one interruption, and the total number of nonsleeping windows forms the total duration (multiplied by the window size of 5 minutes). Both features are only calculated within the interval of BedTime – WakeUpTime, also known as the SleepTime.

As we did not know the validity of BedTime and WakeUpTime—based on which all features discussed so far—we added a feature called TotalSleep that is simply the number of sleeping windows at night (10 PM-10 AM) multiplied by the window size (ie, 5 minutes). Another feature not based on sleep or wake-up times but can be useful is the physical activity of the previous day, as measured by AAR. Concretely, UserActive is the sum of the time spent walking or cycling during the previous day.

### Modeling Approach

As the purpose of this research was to predict SSQ, which cannot be measured directly, we used proxy measures to validate our approach. In particular, we trained models to predict the SSQ reported each morning, average NA of the next day, and the participants’ general level of depressive symptoms. All these variables are on a scale of 0 to 100. The input features used were those described in the previous section (ie, SleepTime, devAvgBedTime, devAvgWakeUpTime, nInterruptions, InterruptionsDuration, UserActive, and TotalSleep). For better understanding the effect of independent variables on dependent variables, only 1 input feature (eg, SleepTime) per dependent variable was used. Therefore, 21 combinations of independent and dependent variables had to be tested. We also attempted using several models to see which one worked best. We applied multilevel models (MLMs) [32], k-nearest neighbors (kNN) [33], support vector machines (SVMs) with a radial kernel [34], extreme gradient boosting (XGB) [35], and generalized additive models (GAMs) with smoothing splines [36]. For predicting depression, linear models (LMs) were used instead of MLMs because these data do not have a nested structure (ie, every participant has only 1 data instance). All these models were trained and tuned using the caret package in the R statistical software package [37]. Because there were several missing sleep values (see the Results section), we decided to apply only generalized and not personalized models because these would be left with too few observations.

### Outlier Analysis

Because of the noisy nature of this type of data, outlier analysis may be needed to remove unlikely observations. Several strategies are available to detect outliers, including a strategy where no outliers are removed. A simplistic method for doing this is by subtracting a multiple of the SD from the median. First, the median was chosen because some variable distributions may be significantly skewed, and secondly, we chose to multiply the SD by 3 because we only wanted to throw out those observations that were truly outliers. A slightly adjusted version of this method is one that does not use the SD but uses the MAD because this is often a more robust measure of dispersion. Finally, a multivariate outlier method was also applied, namely isolation forests that work by building a decision tree to isolate anomalies [38,39]. Outlier observational strategies were also parameters evaluated in this multiverse analysis and are given in Table 2.

### Multiverse

As mentioned earlier, an important aim of this study is to evaluate the effect of several data preprocessing, sensor, and modeling choices on the prediction of sleep quality, mood, and depression via a multiverse study. Table 2 gives an overview of all the multiverse variables.

**Table 2:** Multiverse parameters and values considered in this study^a^.

Parameter			Number of values (N)
**Sensors**			10
	Sensor thresholds for sensors listed in Table 1		
**Features**			7
	devAvgBedTime		
	devAvgWakeUpTime		
	InterruptionsDuration		
	nInterruptions		
	SleepTime		
	TotalSleep		
	UserActive		
**Outcome variables**			3
	Subjective sleep quality		
	Negative affect		
	Depression		
**Models**			5
	Generalized additive models with smoothing splines		
	k-nearest neighbors		
	Mixed effects models		
	Support vector machines		
	Extreme gradient boosting		
**Outlier removal**			4
	None		
	median(x) ± 3σ_x_ per participant		
	median(x) ± 3 × mad^b^(x) per participant		
	Isolation forests; Liu et al [38]; Cortes [39]		
**Feature thresholds**			3
	3 to 5 adjacent windows		
Total number of combinations			12,600

^a^For each parameter, N indicates how many values are there so that the total number of combinations is the product of N.

^b^mad: median absolute deviation.

Because there are 12,600 combinations in this multiverse setup, overfitting plays an even larger role than usual; that is, simply splitting the data set into a training and test set would lead us to choose the model that overfits the training and test sets the most. Therefore, our workflow to minimize overfitting followed a hold-out set approach given below [40]:

1. Split the data into training, validation, and test sets.
2. Train all models on the training set.
3. Validate these models on the validation set based on *R*^2^.
4. For each outcome variable, choose models (maximum of 5) within 1 SE from the best model (known as the 1-SE rule) [41,42].
5. Test these best models on the test set using *R*^2^.

In this procedure, the training set contains the data of the first week whereas the validation and test sets contain data of days 1 to 4 and days 5 to 7 of the second week, respectively. Alternatively, as we used only 1 data instance per participant when predicting depression, the data set was split such that 60%, 20%, and 20% of the participant data used for the training, validation, and test sets, respectively, were stratified by gender. By doing this, we extracted only the best models based on the validation set and then used them on the test set to confirm if they overfitted the test set. Results were measured by their predicted *R*^2^ [43], a performance metric that is calculated as follows:

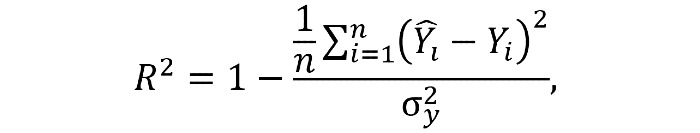



where *n* represents the number of data points, *Y* is the vector of the observed values, and is the vector of the predicted values. In essence, this representation of *R*^2^ is a scaled version of the commonly used mean squared error when applying machine learning models or cross-validation.

## Results

### Missing Data

An important consideration in this type of data is missingness. There are multiple reasons for this. First, participants were prompted with only 14 beeps asking about SSQ in total, so missing 1 beep could have led to the loss of valuable data. Second, preprocessing and outlier removal techniques are responsible for deleting even more data. This coincides with a third major reason for missing data; the wake-up time was often not found for several sensors. This is especially true for the light sensor possibly because it could not detect enough instances where the illuminance was higher than the threshold because the threshold was too high, the phone was upside down, or the phone disappeared inside the participant’s pocket quickly after waking up; there could be other reasons as well. In our analyses, missing data were not imputed but considered as missing at random.

### Descriptive Statistics

**Figure 2:**
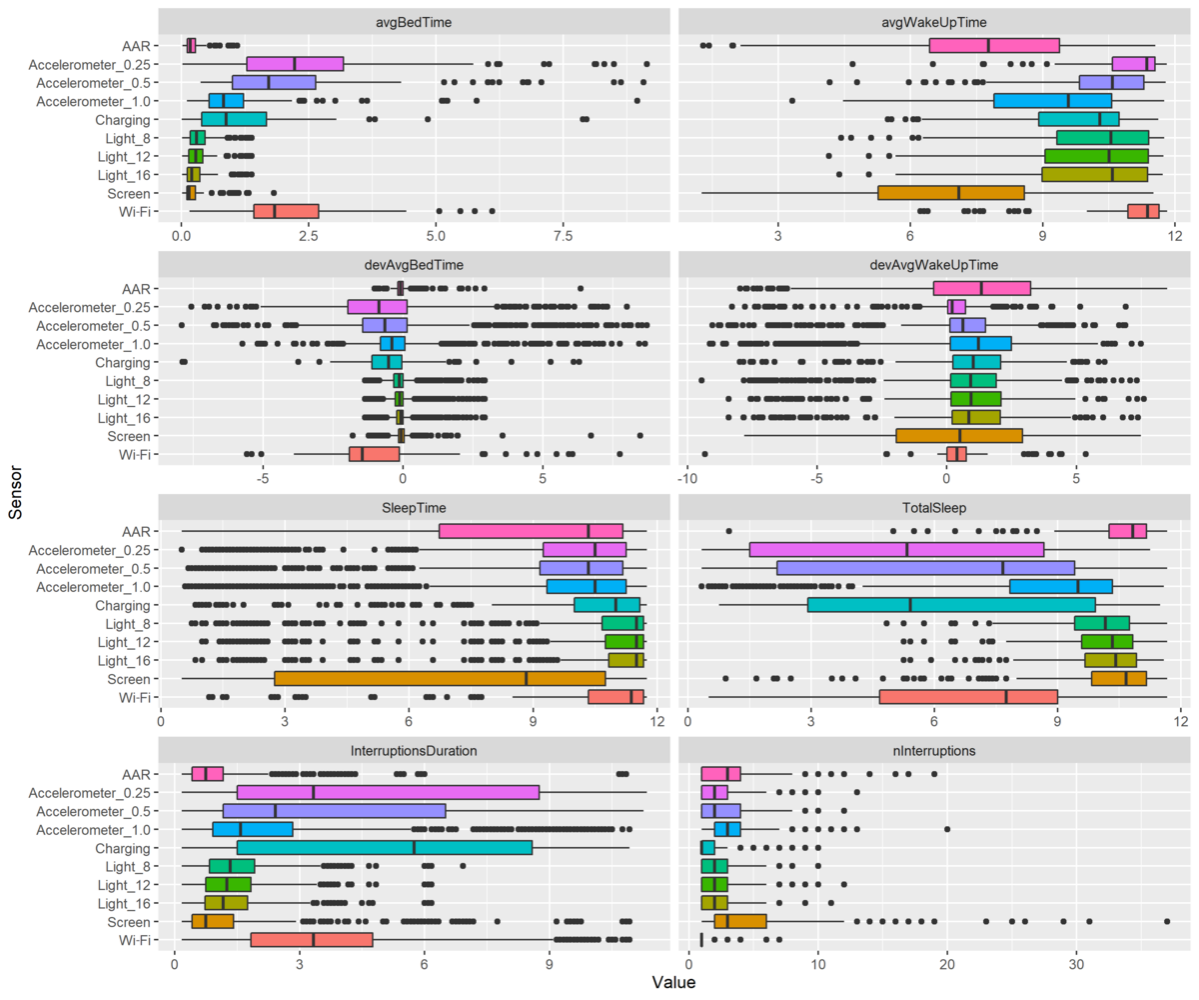
Boxplots showing how different settings in this multiverse study (for parameters see Table 2) affect each feature per sensor. The smaller the range of the boxplot, the more robust a feature is to multiverse choices. The feature for physical activity performed the previous day (UserActive) is not shown because this value does not vary with any multiverse parameter except in outlier analysis. Observe that the x-axis shows hours except in the case of nInterruptions, which shows a count. Moreover, avgBedTime and avgWakeUpTime are influenced by the criterion for defining a night, namely from 10 PM to 10 AM.

To obtain a better understanding of what the features encompass, Figure 2 presents boxplots for each feature per sensor. The displayed variation can be interpreted as how the values of the features change based on what settings (excluding the prediction model choices) are chosen for the multiverse parameters. Therefore, this variability is not related to the variability of features over participants; it purely highlights the robustness of the features with regard to the multiverse choices.

In general, the values within the features in Figure 2 vary considerably, depending on the sensors and other multiverse parameters. There are many outliers that can either represent true patterns or that occur because of data anomalies. Because of the explorative nature of this study and the many combinations we attempted, we assumed the actual patterns to be restricted to the boxes and whiskers, with outliers representing rare behavior or, more likely, data errors. We will now describe some of the key observations and patterns regarding specific features.

First, notice that devAvgBedTime and devAvgWakeUpTime are skewed toward 0. This occurs because these features are the deviations from the mean bedtime or wake-up time. Deviations for bedtime are usually not too high, but for the wake-up time, they seem more problematic. For bedtime, the range for some sensors is very small and skewed toward 0 (ie, at 10 PM), indicating that on average, participants went to bed as soon as the night started or possibly even earlier, no matter what values the multiverse parameters have. On the other hand, the spread for wake-up time is rather large, indicating that changing multiverse parameters substantially impacts this feature. A second observation is that SleepTime appears to be more stable than TotalSleep, although this is not true for every sensor (eg, screen state).

One of the aims of conducting this multiverse experiment was to consider different thresholds for the light sensor and accelerometer. The light sensor provides consistent results for most features (ie, less spread), but this is mostly because almost all participants’ bedtime is immediately at 10 PM. In other words, the highest variability appears to stem from a fluctuating wake-up time. For the accelerometer, a lower threshold corresponds to a later bedtime, a later wake-up time, and longer nightly interruptions. Furthermore, SleepTime is similar between different light and accelerometer thresholds, whereas TotalSleep is vastly different for the accelerometer but not for light.

### Correlations

**Figure 3:**
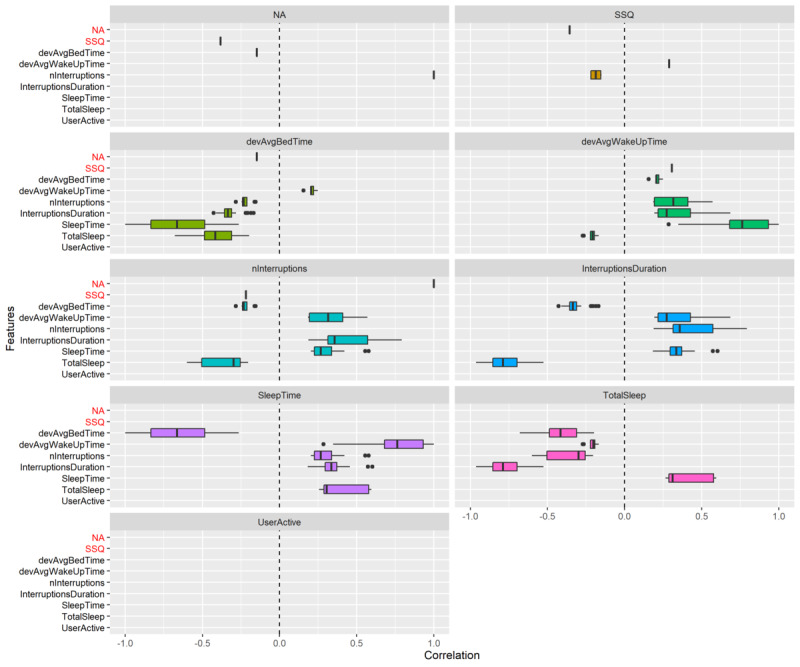
Distribution of significant correlations between variables on the y-axis and in the facets. Note that in contrast to Figure 2, this plot does not show AvgBedTime and AvgWakeUpTime because there is only a single value per participant. SSQ and NA (highlighted in red) are outcome variables denoting the subjective sleep quality and negative affect, respectively. The variance in the boxplots represents how the correlation changes with different values for the multiverse parameters (including different sensors). Without the boxplot, there was no significant correlation. This plot is symmetrical, meaning that every relationship is also shown oppositely. Note that when the boxplot is reduced to a single bar, this likely means there is only a single value (or less likely that there are multiple values very close together).

To better understand how different features relate to each other, Figure 3 shows significant correlations between features, between outcome variables, and between features and outcome variables. Correlations were extracted from mixed effects models as the coefficients of fixed effects using standardized inputs. The *P* values were calculated using analysis of variance. All correlations have been corrected for multiple tests [44]. Figure 3 shows that significant correlations between variables are scarce; for instance, UserActive is not related to any other variable and the outcome variables NA and SSQ are also sparingly related to other variables. In fact, NA is only related to SSQ and the number of interruptions (nInterruptions) whereas SSQ is also only related to NA, nInterruptions, and the deviation from the average wake-up time (devAvgWakeUpTime).

For depression, correlations were not extracted from an MLM (and hence not shown in Figure 3) but rather as Pearson correlations because there is only a single observation per participant. For all variables, the mean was used to generate correlations with depression scores, and multiple testing correction was subsequently applied [44]. There was 1 significant negative correlation with depression and the feature SleepTime (*r*=−0*.*46) using the accelerometer (1 m/s^2^), and 7 negative correlations with the outcome variable SSQ (*r* =−0.46 to −0*.*51) (0.25 to 1 m/s^2^). Furthermore, there was 1 significant positive correlation between depression and NA (*r*=0*.*51) using AAR.

### Evaluation

As described previously, the data set was divided into training, validation, and test sets. To gain a better understanding of how each sensor and sleep feature relates to the output variables, we trained separate models for each feature, sensor, and model combination. By doing so, we could inspect their performance (measured in terms of *R*^2^) on the validation set in relation to the other multiverse parameters. After doing so, we selected the best (maximum 5) models within 1 SE from the best model (ie, the model with the highest *R*^2^ on the validation set) and tested them on the test set to draw definitive conclusions.

**Figure 4:**
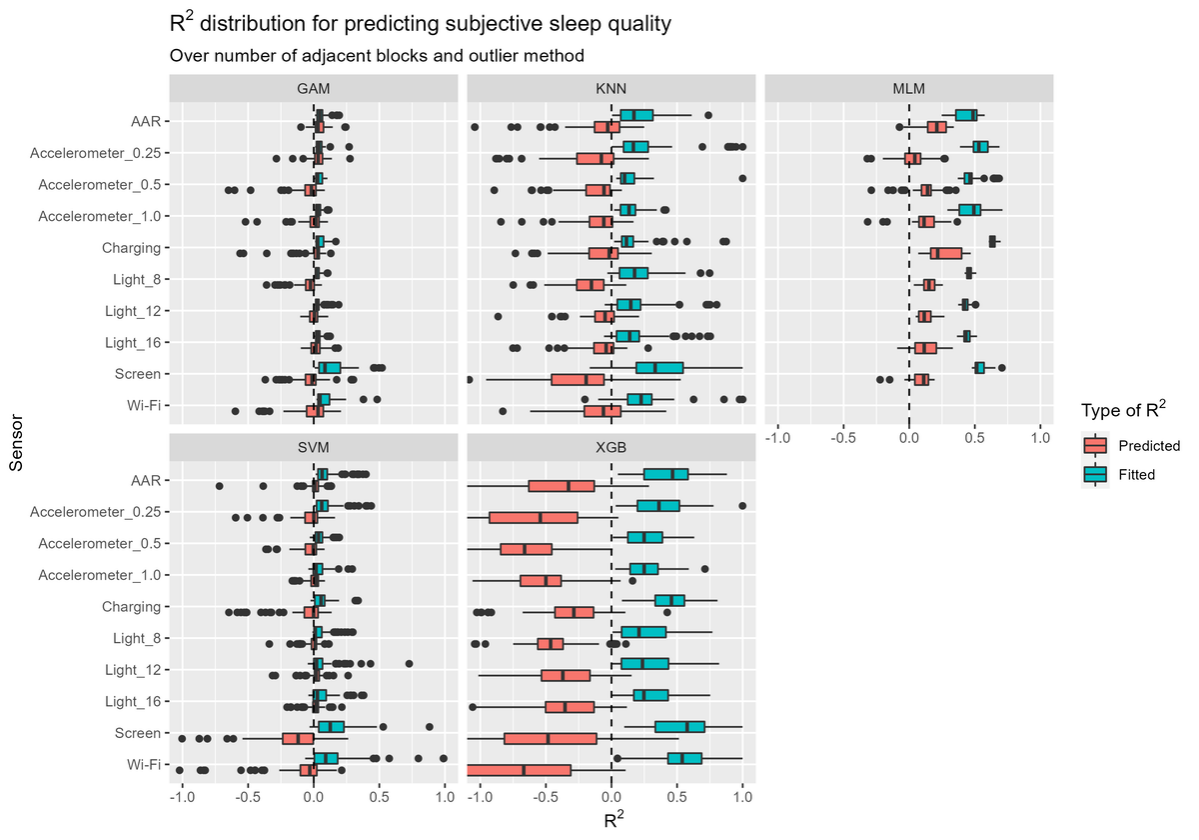
"R2" distribution for predicting subjective sleep quality. The spread of the boxplots represents how the "R2" of predictions of subjective sleep quality change with varying multiverse parameters, namely different sensors, features, models, number of adjacent windows, and outlier methods. GAM: generalized additive model; kNN: k-nearest neighbors; MLM: multilevel model; SVM: support vector machine; XGB: extreme gradient boosting.

First, we present the results of our predictions of SSQ on the validation set in Figure 4, where we can see the predictive performance of different models and the impact of selecting a specific sensor varying with other multiverse parameters (as specified in Table 2). In this plot, it is clear that the MLMs perform the best and the XGB models perform the worst in terms of the predicted *R*^2^ on the validation set. Moreover, the GAMs and SVMs perform similarly, having an *R*^2^ of approximately 0 with extreme values on either side. The kNN models show a slightly poorer performance, usually with an *R*^2^ below 0. In terms of more general patterns, we can see that the models have generally overfitted the data; that is, the models have achieved a higher *R*^2^ on the training set than on the validation set. However, the boxes and whiskers of the boxplots represent only the majority of the results, and even a single outlier may represent a model that does not overfit and performs well. After all, the best models that we selected to be tested on the test set are probably “outliers” in these figures. As far as sensors are concerned, the charging sensor seems to perform slightly better, at least when using MLMs or XGB models but not for others. The other sensors appear to be approximately equivalent in performance. As observed in Multimedia Appendix 1, we replaced the sensors on the y-axis with features so that we could obtain an idea of how different features affect the outcome. As these boxplots are also separated based on the models used, they have approximately the same *R*^2^ distribution, as shown in Figure 4. The performances of features are relatively similar. One might argue that nInterruptions generally performs slightly better in combination with XGB models and MLMs, but this effect is small.

**Figure 5:**
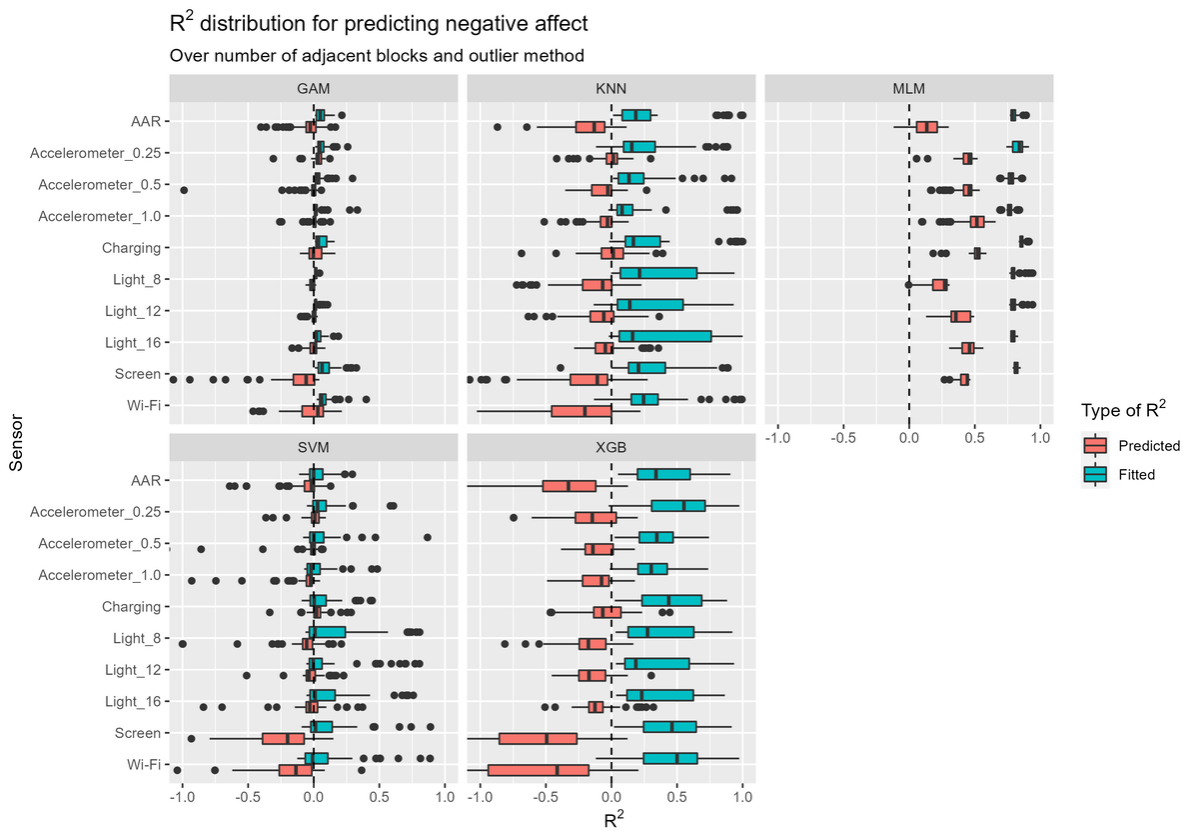
"R2" distribution for predicting negative affect. The spread of the boxplots represents how the "R2" of predictions of negative affect change with varying multiverse parameters, namely different sensors, features, models, number of adjacent windows, and outlier methods. GAM: generalized additive model; kNN: k-nearest neighbors; MLM: multilevel model; SVM: support vector machine; XGB: extreme gradient boosting.

Second, the predictive performance regarding NA on the validation set is represented visually in Figure 5. Again, for most models, the MLMs appear to perform best as they show the highest *R*^2^ on the validation set. Nevertheless, as indicated by the high discrepancy in *R*^2^ values between the training and validation set, models are once again overfitting. Similar to that observed in the prediction of SSQ, the XGB models perform the worst followed by the kNN models, and finally GAMs and SVMs, showing a predictive *R*^2^ value of approximately 0. A notable difference among these models is that there is little variation among the MLMs, GAMs, and SVMs, meaning that multiverse parameter choices have less impact than that observed for the kNN and XGB models.

When predicting NA, choosing a specific sensor does not seem to matter, except for screen activity and Wi-Fi because these performed much worse. Indeed, all MLMs using the Wi-Fi sensor were found to be inoperable, indicating a serious problem with these models (eg, not enough data, no convergence). Compared to the rest of the sensors, screen activity and Wi-Fi also performed much worse when using XGB models. When finding the best performing feature, Multimedia Appendix 2 shows a trend similar to that of the sensors, meaning that there is no single feature that is better than all the rest.

**Figure 6:**
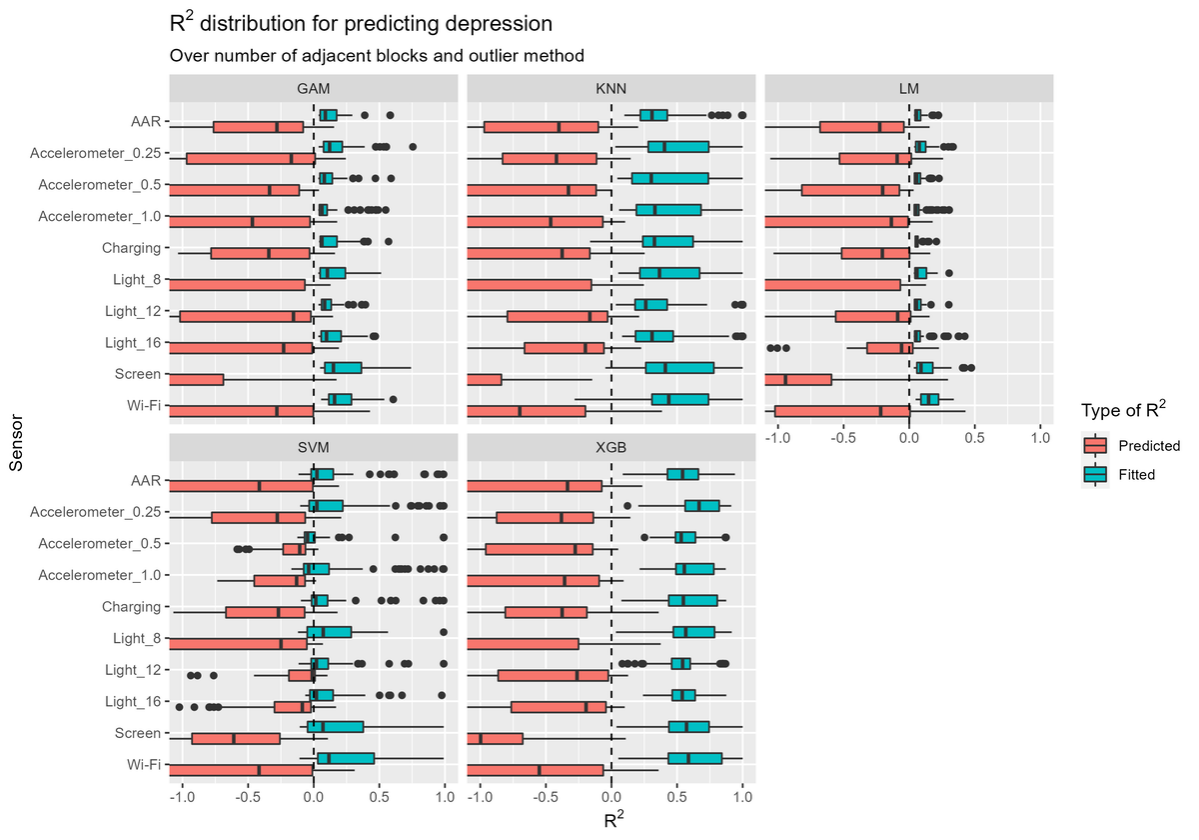
"R2" distribution for predicting depression. The spread of the boxplots represents how "R2" values when predicting depression change with varying multiverse parameters, namely different sensors, features, models, number of adjacent windows, and outlier methods. GAM: generalized additive model; kNN: k-nearest neighbors; MLM: multilevel model; SVM: support vector machine; XGB: extreme gradient boosting.

Finally, Figure 6 shows the performance distribution when predicting depression in relation to other multiverse parameters. Contrary to SSQ and NA, the model performance spread in terms of *R*^2^ for depression is much greater, implying that the selection of the multiverse parameters influences model performance. There are considerable differences among sensors too. For example, charging and AAR appear to perform better across all models than the other sensors; that is, because their spread is smaller, models using the charging or AAR sensor are likely to perform better. On the other hand, the 1 m/s^2^ accelerometer has a much larger spread and a lower mean *R*^2^ than the other sensors.

Multimedia Appendix 3 presents similar findings about the features when predicting depression. That is, the predictions are much worse than those for other outcome variables. The nInterruptions feature appears to perform slightly better than the others although this applies only to the spread, implying that with this feature, models perform better on average but that there may be models from other features performing much better.

**Figure 7:**
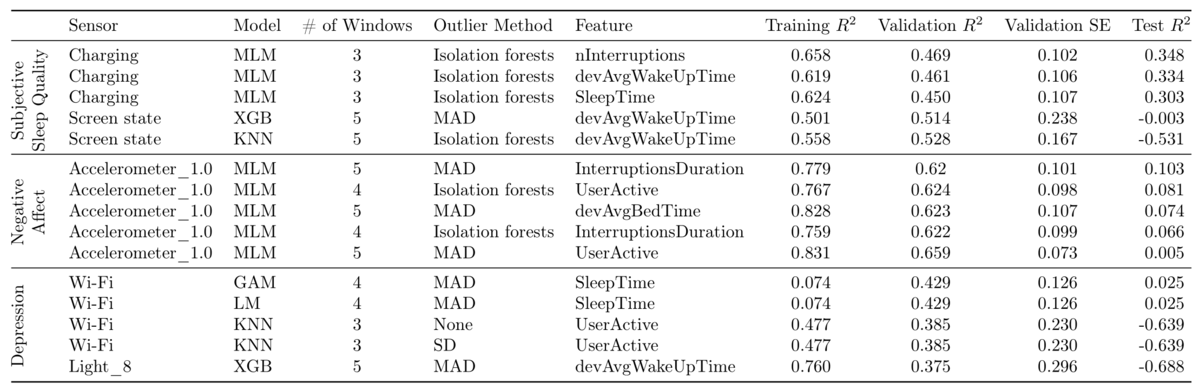
"R2" distribution for predicting depression. The spread of the boxplots represents how "R2" values when predicting depression change with varying multiverse parameters, namely different sensors, features, models, number of adjacent windows, and outlier methods. GAM: generalized additive model; kNN: k-nearest neighbors; MAD: median absolute deviation; MLM: multilevel model; SVM: support vector machine; XGB: extreme gradient boosting.

Although it is important to describe how different sensors and features impact the outcome variables on average, our ultimate objective is to find how we can best predict the outcome variables from the available data. To this end, Figure 7 presents the results of the best models per outcome variable. Unsurprisingly, the sensors and features of these best models are generally also the ones that performed the best on average for those outcome variables. However, there is a remarkably large gap between the *R*^2^ values on the test set and those on the training and validation sets. For example, a kNN (k=7) model for predicting SSQ using the screen state sensor, 5 adjacent windows, isolation forests, and the feature devAvgWakeUpTime achieved *R*^2^ values of 0*.*558 and 0*.*528 on the training and validation sets, respectively. As this does not show much overfitting, one would expect the test *R*^2^ to be similar. However, the *R*^2^ on the test set is only −0*.*531, revealing the model to be less than informative.

Figure 7 shows that SSQ can be predicted reasonably well; MLMs using the charging sensor, 3 adjacent windows, and isolation forests for detecting outliers achieved a test *R*^2^ of 0.303 to 0.348 with nInterruptions, devAvgWakeUpTime, or SleepTime. All these features were positively related to SSQ, having a slope of approximately 0.442–0.643 with a high 95% CI –3.06 to 4.35. However, predicting NA proved to be more challenging because we obtained a highest *R*^2^ of only 0.103 on the test set, a value that is considerably lower than that obtained on the training and validation sets. Nevertheless, it is worth noting that all the best models for predicting NA are MLMs using the accelerometer with a threshold of 1 m/s^2^. Of these models, the ones using InterruptionsDuration and devAvgBedTime were positively related to NA (slopes of 0.0449-0.279 and 0.427, respectively) whereas userActive was negatively related (slope of 0.199-1.37). Finally, predicting depression was the most difficult task because only 1 best model yielded a barely informative result, namely the LM with an *R*^2^ of 0.025. The predictions for SSQ appear better because it is more closely associated with objective sleep than the other outcome variables, namely NA and depression.

## Discussion

### Principal Findings

Although various studies have used different methods and sensors to successfully track sleep with mobile sensing [14,25,26], it is not yet clear how these various approaches impact the results. To this end, this study was designed to provide a transparent and robust method for tracking sleep with mobile sensing. By accounting for many choices in a multiverse study design, we were able to separate and elucidate the various effects of sensors, features, and models on sleep proxy measures, thereby providing an important step forward for future researchers. Principally—and in accordance with previous research—we showed that it is possible to explain a reasonable and an interesting proportion of the variance observed in SSQ (or the related variable of daily negative mood) using mobile sensing. Moreover, our multiverse analysis indicates which combinations of multiverse parameters (shown in Table 2) can do this best, at least in this sample. Although the results in this study are slightly more pessimistic than those in other studies concerning mobile sensing and sleep (ie, some studies showing very high accuracies) [12,25,26], they cannot be compared directly given that we applied a strict cross-validation plan.

From the outlined results, we can conclude that for predicting SSQ, the charging sensor is overall the best performing and the most robust sensor although this sensor has a significant advantage because participants were instructed to leave their smartphone charging at night. However, for predicting NA, we found the accelerometer to be the best sensor despite its previously noted limitations (ie, low sample rate and measurements only along the x-axis). Based on previous research [11,14], this finding suggests that the accelerometer is a feasible option for sleep detection. Nevertheless, when predicting person-level depression, we found that the results varied considerably and were often less than informative. Even the best performing sensor achieved an *R*^2^ of only 0.025 on the test set, and hence we could not draw any conclusions from this.

Another aspect we accounted for in this study is using multiple ways of constructing models (ie, we attempted multiple models to see which one worked best). The results point out that MLMs are clearly the best performing models for any outcome variable. However, it should be noted that the analysis is between participants whereas MLMs also use within-person information (although large amounts of within-participant data were missing). Likewise, LMs seem to be the primary choice for predicting depression as well, suggesting that robustness must be an important characteristic. Furthermore, GAMs and SVMs showed reasonably similar performances but much worse than that of MLMs. Finally, kNN and XGB models seemingly performed the worst, with XGBs displaying excessive overfitting.

Although not described in detail, Multimedia Appendix 1 presents the *R*^2^ distributions of the results split by feature. We have already mentioned that no feature appears to be more important than any other. Another inference supported by Figure 2 is that some features are more robust than others in this multiverse analysis. Concretely, nInterruptions has less spread than the other features, indicating that it is a relatively robust feature. This could be interesting for future studies in which researchers have to manually select features.

### Limitations

There are a number of limitations that should be considered when interpreting the results of this study. The results of this study are generally limited by the relatively small size of the data set. This limitation poses a drawback because it makes the models more prone to overfitting, although this risk was mitigated by an elaborate hold-out set approach. Next, we list 3 distinct limitations that may have impacted the results.

First, we relied on self-reports from ESM as an estimate of sleep. This, combined with the fact that participants did not always answer the SSQ item in the morning, may have led to odd or spotty patterns. Despite trying to minimize the impact of this factor by predicting several outcome variables known to be associated with sleep (ie, not only SSQ but also NA and depression), we cannot be certain about the impact of this factor on the research results.

The second limitation resulted from several sensor issues during the trial. The most pressing issue is the low sample rate for the accelerometer, charging, light, and Wi-Fi, averaging approximately 1 measurement instance every 2.9 minutes. Especially for the accelerometer, this is not enough data because it only allows the sensors to record momentary changes instead of tracking participants’ contextual surrounds for longer periods, as mentioned earlier. The accelerometer also did not collect the y- and z*-*axes data, which further limits its use. Furthermore, the way in which sensors collected data was directly influenced by participants’ behavior in that they were instructed to keep the smartphone charged at night and be connected to Wi-Fi. This may mean the charging and Wi-Fi sensors are less ecologically valid.

Finally, we assumed that a normal night of sleep is somewhere between 10 PM and 10 AM the next day. Although this assumption is probably true for most people, students may not always follow a regular sleep schedule. Moreover, because some sensors are prone to produce many false positives (eg, the light sensor detecting the smartphone being in a participant’s pocket as sleep), we felt it was necessary to reduce this at the cost of cutting off a small portion of the participants’ sleep.

### Contributions to Previous Work

Our work complements previous work described in various ways. First and foremost, this study developed and implemented a method to unravel the effects of distinct variables as well as sensors and their thresholds for the prediction of SSQ, NA, and depression. Such a method can guide future researchers intending to conduct similar studies to further examine these effects or help them choose which sensors to select for estimating sleep. It also provides a tool for researchers when they have to make choices based on the many different results that exist when predicting sleep with mobile sensing, such as the widely varying results in earlier studies [22-26]. Moreover, it stresses the importance of making considerations and makes researchers aware that such choices may have a significant impact on their study, as suggested by the multiverse theory [27] and previous studies [26]. In addition, this study can help verify theories that link smartphone-recordable behaviors to sleep and mood, such as the purported links among sleep, depression, and stress [19].

Second, we showed that continuous SSQ and NA can be reasonably predicted from sleep features whereas depression cannot be predicted. Most studies reduce these features to a discrete variable with only 1 or 2 categories [12,22], whereas we took on the challenge to expand this to a continuous variable between 0 and 100. Naturally, a continuously scaled variable brings about more variation, but this variation can be approximated.

### Future Directions

When performing such an explorative study where it is not yet clear how different variables relate to each other, it is important to at least have enough data to work with. Future studies should focus on obtaining a sufficiently large sample size to minimize the necessity of a potential multiverse study or at least minimize the risk of selecting a nonrepresentative sample. Additionally, missing data posed a big challenge in this study because participants did not respond to the first beep of the day.

Although this study has solely relied on SSQ and proxy measures such as NA and depression, in future studies, it could be useful to also collect self-reported bed- and wake-up times to validate these features. After all, several other features were based on these times; therefore, obtaining a better understanding of their accuracy also helps in further improving these features. Moreover, wearable devices are not as reliable as PSG, but they are considerably less obtrusive and have already been adopted by a sizable portion of the mainstream population to record sleep. As such, they can be added in future studies to achieve an additional high-quality estimate of sleep. Finally, if an approach could be developed to use mobile sensing data to estimate sleep (eg, through a multiverse approach), it should be compared to PSG as a final measure of validation.

### Conclusions

Sleep plays an important part in moods and mood disorders but is difficult to track unobtrusively. Therefore, this study has shown that it is feasible to track sleep with mobile sensing although this depends strongly on which sensors, features, and models are chosen. In fact, our approach demonstrated that most combinations of multiverse parameters often form noninformative models. This can be partly attributed to issues with data collection and not having enough data for developing personalized models, but further research is needed to validate these problems. Moreover, SSQ predictions were better than the other proxy measures (ie, NA and depression severity). Nevertheless, the findings of this study are promising and warrant further investigation into the use of mobile sensing for tracking sleep.

## Appendix

Multimedia Appendix 1 "R2" distribution for predicting subjective sleep quality. The spread of the boxplots represents how features change with varying multiverse parameters, namely different sensors, number of adjacent windows, and outlier method.

Multimedia Appendix 2 "R2" distribution for predicting negative affect. The spread of the boxplots represents how features change with varying multiverse parameters, namely different sensors, number of adjacent windows, and outlier method.

Multimedia Appendix 3 "R2" distribution for predicting depression. The spread of the boxplots represents how features change with varying multiverse parameters, namely different sensors, number of adjacent windows, and outlier method.

## Abbreviations

AAR: Android activity recognition
ESM: experience sampling method
GAM: generalized additive model
kNN: k-nearest neighbors
LM: linear model
MAD: median absolute deviation
MLM: multilevel model
NA: negative affect
PSG: polysomnography
SSQ: subjective sleep quality
SVM: support vector machine
XGB: extreme gradient boosting
