# Supplementary figures and images for: Tracking Subjective Sleep Quality and Mood With Mobile Sensing: Multiverse Study
Source: J Med Internet Res. 2022 Mar 18;24(3):e25643. doi: 10.2196/25643 (PMC8976254; doi:10.2196/25643)

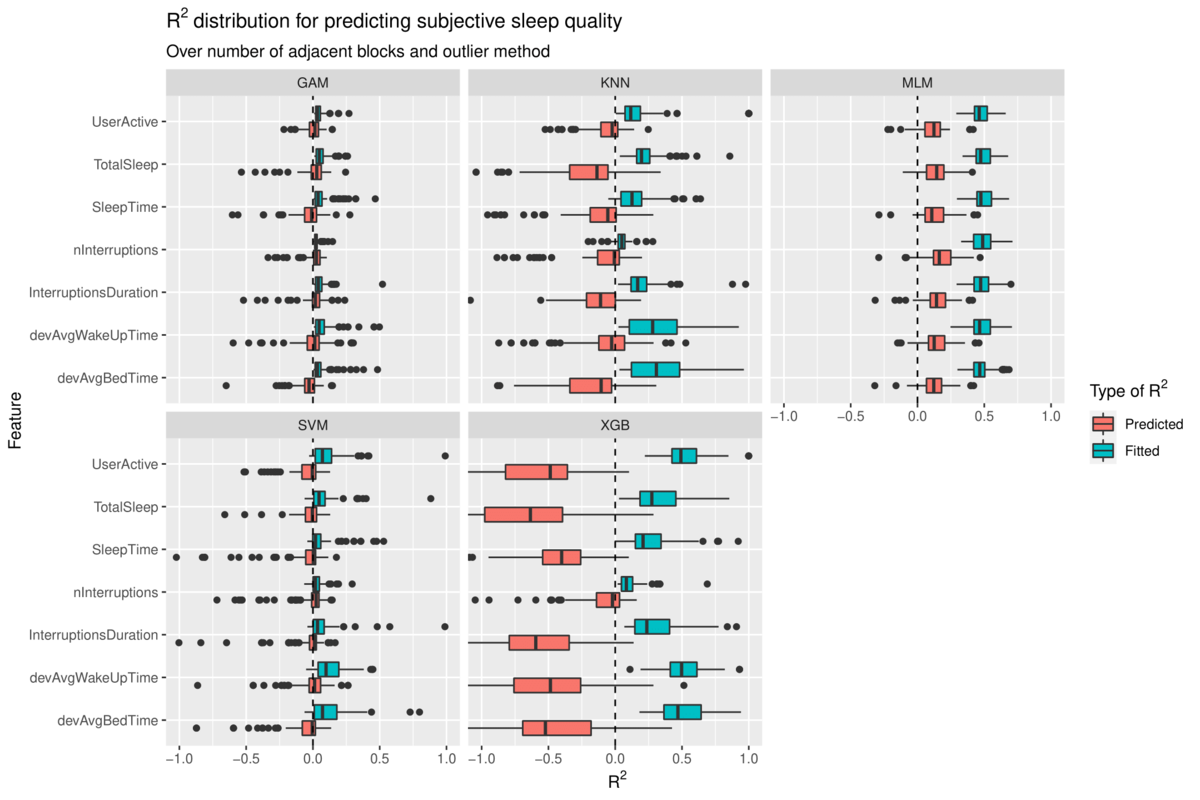

Supplement: Multimedia Appendix 1 [file jmir_v24i3e25643_app1.png]

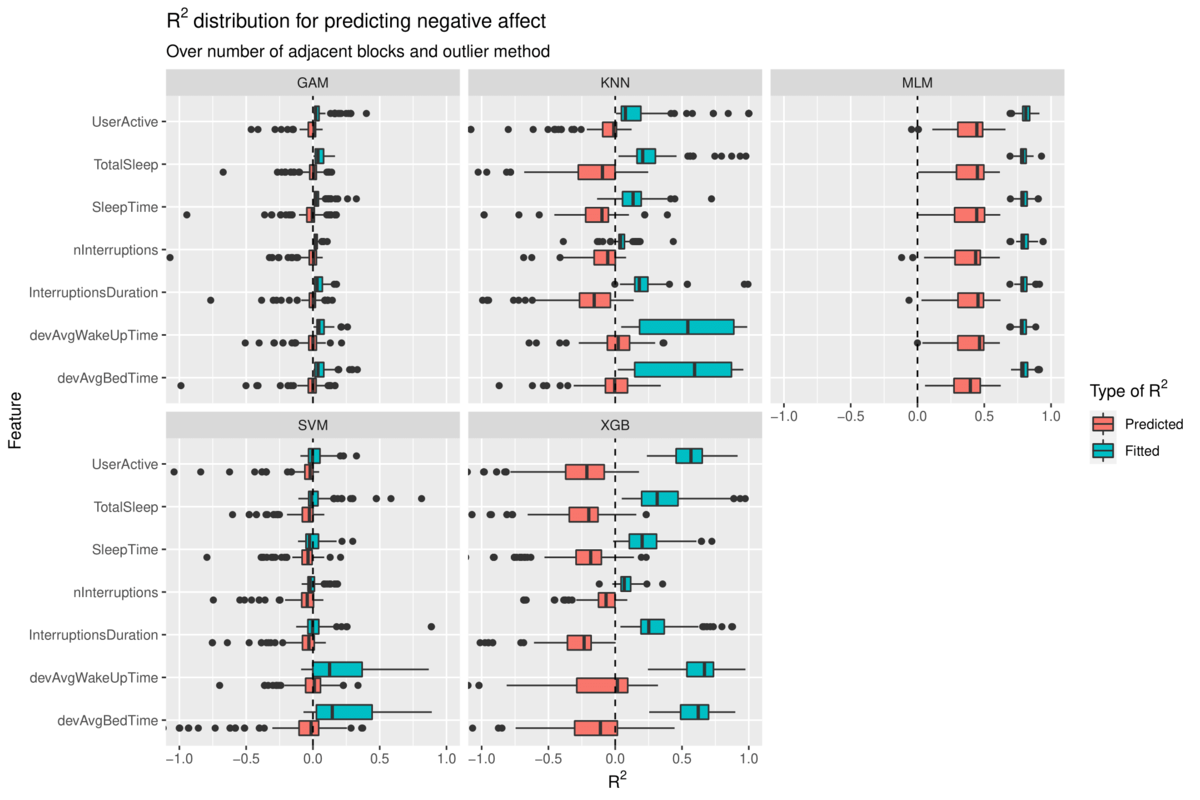

Supplement: Multimedia Appendix 2 [file jmir_v24i3e25643_app2.png]

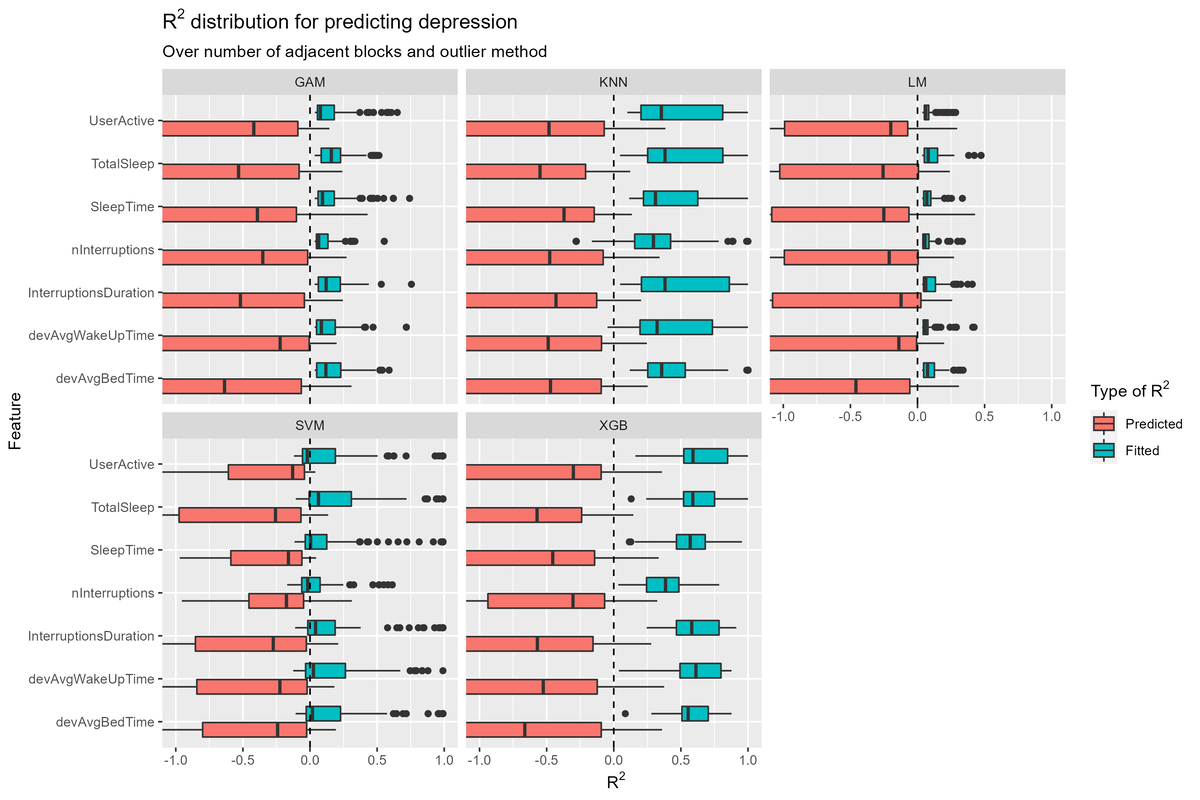

Supplement: Multimedia Appendix 3 [file jmir_v24i3e25643_app3.png]
